# MetaNN: accurate classification of host phenotypes from metagenomic data using neural networks

**DOI:** 10.1186/s12859-019-2833-2

**Published:** 2019-06-20

**Authors:** Chieh Lo, Radu Marculescu

**Affiliations:** Department of Electrical and Computer Engineering, Carnegie Mellon University, 5000 Forbes Ave., Pittsburgh, PA, USA

**Keywords:** Metagenomics, Neural networks, Host phenotypes, Machine learning

## Abstract

**Background:**

Microbiome profiles in the human body and environment niches have become publicly available due to recent advances in high-throughput sequencing technologies. Indeed, recent studies have already identified different microbiome profiles in healthy and sick individuals for a variety of diseases; this suggests that the microbiome profile can be used as a diagnostic tool in identifying the disease states of an individual. However, the high-dimensional nature of metagenomic data poses a significant challenge to existing machine learning models. Consequently, to enable personalized treatments, an efficient framework that can accurately and robustly differentiate between healthy and sick microbiome profiles is needed.

**Results:**

In this paper, we propose MetaNN (i.e., classification of host phenotypes from *Metagenomic* data using *Neural Networks*), a neural network framework which utilizes a new data augmentation technique to mitigate the effects of data over-fitting.

**Conclusions:**

We show that MetaNN outperforms existing state-of-the-art models in terms of classification accuracy for both synthetic and real metagenomic data. These results pave the way towards developing personalized treatments for microbiome related diseases.

## Background

Due to recent advances in modern metagenomic sequencing methods, several studies have characterized and identified different microbiome profiles in healthy and sick individuals for a variety of microbiome related diseases. For example, for the inflammatory bowel disease (IBD) which affects approximately 1.8 million Americans, it has been shown that individuals have about (30-50)% less biodiversity of commensal bacteria (e.g., *Firmicutes* and *Bacteroidetes*) compared to healthy individuals [1]. Another example is the Type 2 diabetes (T2D) which affects approximately 29.1 million Americans and costs the healthcare system about 245 billion dollars annually. T2D patients show significant changes in the 190 operational taxonomic units (OTUs) (OTU is defined as groups of closely related microbes.), particularly a high abundance of *Enterobacteriaceae* compared to a healthy control group [2]. As a consequence, such differences in the microbiome profiles can be used as a diagnostic tool to differentiate the disease states of an individual. Being able to accurately differentiate the disease states for an individual can ultimately pave the way towards precision medicine for many microbiome related diseases.

A common and widely used approach to characterize the human microbiome profile relies on using the 16S rRNA gene as the taxonomic maker. Indeed, based on this profiling technique, previous studies have used unsupervised learning techniques such as clustering and principal coordinates analysis (PCoA) to perform classical hypothesis testing in order to classify microbial samples [3]. However, these methods are limited in their ability to classify unlabeled data or extract salient features from highly complex or sparse data; consequently, many supervised learning methods have been designed specifically for such classification purposes. For instance, several studies have shown that one can successfully identify differences in the microbiome profile or function of different host phenotypes such as body site, subject, and age [4, 5].

In terms of classification methods, machine learning (ML) models are powerful tools for identifying patterns in highly complex data, including human metagenomic data. In particular, supervised learning methods have been widely used for classification tasks in different areas such as image, text, and bioinformatics analyses [5]. For a typical supervised classification task, each training data point (sample) consists of a set of input features (e.g., relative abundance of taxa) and a qualitative dependent variable giving the correct classification for that data point. For example, microbial samples from human body sites may be labeled as gut, mouth, or skin [6]. The goal of supervised learning is then to develop predictive models (or functions) from training data that can be used to assign the correct class (or category) labels to new samples.

Challenges of host phenotypes classification stem from the very nature of the high dimensionality of the metagenomic data. For instance, a typical dataset may contain few hundred samples, but thousands of OTUs (i.e., features); this large number of features can greatly challenge the classification accuracy of any method and compound the problem of choosing the important features to focus on. Although several ML-based supervised classification algorithms, such as random forest [7], have been successful at classifying microbial samples [5], their classification accuracy remains poor, at least for some datasets [4]. As a consequence, new ML models are needed to improve the classification accuracy.

Recent advances in deep learning have shown significant improvements on several supervised learning tasks such as image classification and object detection [8]. Neural networks (NNs) consist of multiple (non-linear) hidden layers which make them expressive models that can learn complicated relationships between the system inputs and outputs. However, NNs usually require a large amount of training instances to obtain a reasonable classification accuracy and prevent over-fitting of training data. For instance, we need at least tens of thousands of images for a typical image classification task like ImageNet [8]. To the best of our knowledge, we are the first to propose NN models that can be used to classify metagenomic data with small (e.g., in the order of hundreds) microbial sample datasets; this is a challenging problem as the low count of samples can cause data over-fitting, hence degradation of the classification accuracy.

To overcome the problem of data over-fitting, we first consider two different NN models, namely, a multilayer perceptron (MLP) and a convolutional neural network (CNN), with design restrictions on the number of hidden layer and hidden unit. Second, we propose to model the microbiome profiles with a negative binomial (NB) distribution and then sample the fitted NB distribution to generate an augmented dataset of training samples. Additionally, we adopt the dropout technique to randomly drop units along with their connections from NNs during training [9]. Data augmentation and dropout can effectively mitigate data over-fitting as we demonstrate in our experiments and analyses.

Finally, to assess the performance of different ML models, we propose a new simulation method that can generate synthetic microbial samples based on NB distributions which are commonly used to model the microbial count data [10]. As a result, the generated samples consist of distinct microbiome profiles and particular class labels associated with them. To account for the noise in real microbial data, we consider several sources of measurement errors; this can be used to compare the performance of different ML models and identify scenarios that may degrade the classification accuracy significantly.

We test our framework on eight real datasets, i.e., five benchmarks proposed in [5], one example from HMP [6], and two diseases, i.e., inflammatory bowel disease [11] and esophagus [12]. We show that by augmenting the metagenomic data and using the dropout technique during training, the classification performance for the MLP classifier gets significantly better compared to all other existing methods for seven (out of eight) real datasets for two performance metrics commonly used to evaluate classification models: Area under the receiver operating characteristics (ROC) curve (AUC), and F1 score of class label predictions [13].

Taken together, our proposed framework MetaNN (shown in Fig. 1) brings the following three contributions:

1. First, we propose two NN models (i.e., MLP and CNN) for metagenomic data classification based on a new data augmentation method. To the best of our knowledge, we are the first to consider microbial sample augmentation using a statistical method and systematically quantify the performance of NN models against other existing ML algorithms.

**Fig. 1:**
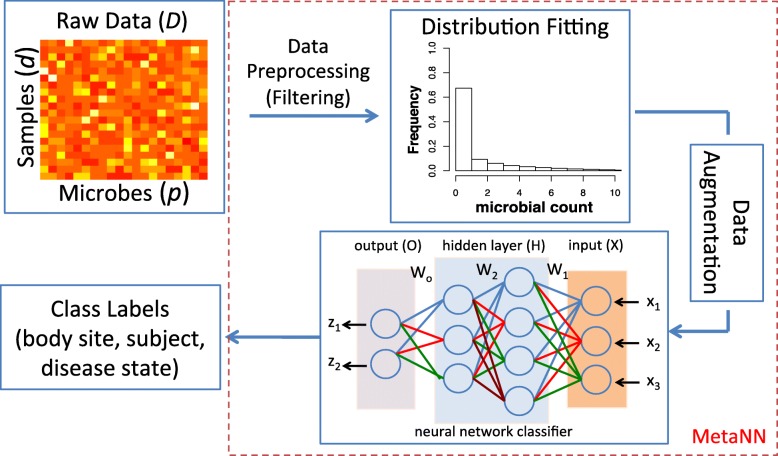
Our proposed MetaNN framework for the classification of metagenomic data. Given the raw metagenomic count data, we first filter out microbes that appear in less than 10% of total samples for each dataset. Next, we use negative binomial (NB) distribution to fit the training data, and then sample the fitted distribution to generate microbial samples to augment the training set. The augmented samples along with the training set are used to train a neural network classifier. In this example, the neural network takes counts of three microbes (*x*_1_,*x*_2_,*x*_3_) as input features and outputs the probability of two class labels (*z*_1_,*z*_2_). The intermediate layers are hidden layers each with four and three hidden units, respectively. The input for each layer is calculated by the output of the previous layer and multiplied by the weights (*W*_1_,*W*_2_,*W*_*o*_) on the connected lines. Finally, we evaluate our proposed neural network classifier on synthetic and real datasets based on different metrics and compare outputs against several existing machine learning models (see Review of ML methods)
2. Second, we propose a new simulation method to generate synthetic data that considers several sources of measurement errors; synthetic data we develop can be freely used by the research community to benchmark classification performance of different ML models.
3. Third, we show that our proposed MetaNN outperforms other models with significant average gains of 7% and 5% in terms of F1-macro and F1-micro scores, respectively.

### Review of ML methods

We compare and contrast different (multicategory) ML classification models: Support vector machines (SVM) [14], regularized logistic regression (LR) [15], gradient boosting (GB) [16], random forest (RF) [7], multinomial Naïve Bayes (MNB) [17] because of their wide and successful application to many datasets from other genomic applications and all the above methods are implemented with scikit-learn (http://scikit-learn.org/stable/) in Python.

Since most of these classifiers are designed for binary classification (i.e., have only two output classes), we adopt a *one-versus-rest* type of approach where we train separate binary classifiers for each class against the rest of data and then classify the new samples by taking a vote of the binary classifiers and choosing the class with the ’strongest’ vote. The *one-versus-rest* type of approach for classification is known to be among the best performing methods for multicategory classification [4].

#### Support vector machines (SVMs)

SVMs perform classification by separating different classes in the data using a maximal margin hyperplane [18]. To learn non-linear decision boundaries, SVMs implicitly map data to a higher dimensional space by means of a kernel function, where a separating hyperplane is then sought. The superior empirical performance of SVMs in many types of high-throughput biomedical data can be explained by several theoretical reasons: SVMs are robust to high variable-sample ratios and large number of features; they can efficiently learn complex classification functions and employ powerful regularization principles to avoid data over-fitting [19].

#### Regularized logistic regression (LR)

LR is a learning method from the class of general linear models that learns a set of weights that can be used to predict the probability that a sample belongs to a given class [18]. Typically, we can add either a *L*_1_ or *L*_2_ penalty to the LR to regularize and select important features. The weights are learned by minimizing a log-likelihood loss function. An *L*_2_ penalty favors solutions with relatively small coefficients, but does not discard any features. An *L*_1_ penalty shrinks the weights more uniformly and can set weights to zero, effectively performing embedded feature selection. We consider both regularizations in our subsequent experiments.

#### Gradient boosting (GB)

GB is a machine learning technique for regression and classification problems which produces a prediction model as an ensemble of weak prediction models, typically decision trees. It builds the model in a stage-wise fashion like other boosting methods do, and then generalizes them by allowing optimization of an arbitrary differentiable loss function; this is achieved by iteratively choosing a function (weak hypothesis) that points in the negative gradient direction.

#### Random forests (RF)

RF is a classification algorithm that uses an ensemble of unpruned decision trees, each built on a bootstrap sample of the training data using a randomly selected subset of features [7]. The RF algorithm possesses a number of appealing properties making it well-suited for classification of metagenomic data: (i) it is applicable when there are more predictors (features) than observations; (ii) it performs embedded feature selection and it is relatively insensitive to the large number of irrelevant features; (iii) it incorporates interactions between predictors: (iv) it is based on the theory of ensemble learning that allows the algorithm to learn accurately both simple and complex classification functions; (v) it is applicable for both binary and multicategory classification tasks; and (vi) according to its inventors, it does not require much fine tuning of hyperparameters and the default parameterization often leads to excellent classification accuracy.

#### Multinomial naïve bayes (MNB)

MNB classifier is suitable for classification with discrete features (e.g., word counts for text classification). Hence, MNB is usually used to classify topics (i.e., class labels) among sentences. For microbial data, a class can contain a mixture of OTUs that is shared among samples. Therefore, we can learn the microbiome mixture conditioned on the class labels.

## Methods

### Acquisition and preprocessing of metagenomic data

In this paper, we utilize the high-quality sequencing reads in 16S rRNA variable regions. The taxonomy (OTU) identification of the 16S rRNA is performed using different pipelines for eight different datasets as summarized in Table 1. The datasets CBH, CS, CSS, FS, FSH are obtained from the study of [5] and originate from the work of [20] and [21]. The HMP dataset is obtained from the high-quality sequencing reads in 16S variable regions 3-5 (V35) of HMP healthy individuals with taxonomy identification done by the QIIME [22] pipeline. The PDX dataset is obtained from [4] and originate from the work of [12].

**Table 1:** Real metagenomic data used in this paper

Dataset	# of samples	# of features	# of classes	Classification task
Classification of body sites				
Costello *et al.* (2009) Body Habitat (CBH)	552	1454	6	Classify body habitats: skin (357), oral cavity (46), External Auditory Canal (44), Hair (14), Nostril (46), Feces (45)
Costello *et al.* (2009) Skin Sites (CSS)	357	600	12	Classify skin sites: external nose (14), forehead (32), glans penis (8), labia minora (6), axilla (28), pinna (27), palm (64), palmar index finger (28), plantar foot (64), popliteal fossa (46), velar forearm (28), umbilicus (12)
Human Microbiome Project (HMP)	1025	323	5	Classify 5 major body sites: anterior nares (269), buccal mucosa (312), stool (319), supragingival plaque (313), tongue dorsum (316)
Classification of subjects				
Costello *et al.* (2009) Subject (CS)	140	464	7	Classify 7 subjects: (20, 20, 20, 20, 20, 20, 20)
Fierer *et al.* (2010) Subject (FS)	104	294	3	Classify 3 subjects: (40, 33, 31)
Fierer *et al.* (2010) Subject x Hand (FSH)	98	294	6	Classify by subject and left/right hand: (20, 18, 17, 14, 16, 13)
Classification of disease states				
Inflammatory Bowel Disease (IBD)	1025	1025	2	Classify disease states: normal (500), IBD (500)
Pei *et al.* (2013) Diagnosis (PDX)	200	5955	4	Classify disease states: normal (28), reflux esophagitis (36), Barrett’s esophagus (84), esophageal adenocarcinoma (52)

We consider three different categories of classification aims: body sites, subjects, and disease states. Number of samples for a particular class is included between the round brackets. The number of features equals the number of different OTUs (i.e., microbes)

The resulting OTU table can be represented by a matrix $D \in \mathbb {N}^{n\times p}$ where $\mathbb {N}$ is the set of natural numbers; *n* and *p* represent number of samples and number of microbes, respectively. $d^{i} = [d_{1}^{i}, d_{2}^{i}, \dots, d_{p}^{i}]$ denote the *p*-dimensional row vector of OTU counts from the *i*^*th*^ sample (*i*=1,…,*n*). The total cumulative count for the *i*^*th*^ sample can be expressed as $s^{i} = {\sum \nolimits }_{k=1}^{p} d_{k}^{i}$. To account for the different sequencing depth of each sample, the raw count data (*d*^*i*^) are typically normalized by the cumulative count (*s*^*i*^) which results in *relative* abundances (or profiles) vector $x^{i} = \left [\frac {d^{i}_{1}}{s^{i}}, \frac {d_{2}^{i}}{s^{i}}, \dots, \frac {d_{p}^{i}}{s^{i}}\right ]$ for any sample *i*. These relative taxonomy abundances are further rescaled in the range [0, 1] and serve as input features for the ML models. Note that the OTU abundance table is constructed without any knowledge of the classification labels and thus data preprocessing does *not* influence the performance of ML models.

### Modeling the microbiome profile

For biological samples, there exist multiple sources (e.g., biological replication and library preparation) that can cause variability of features [10]. In oder to account for such effects, recent work suggests to use the mixture model to account for the added uncertainty [23]. Taking a hierarchical model approach with the Gamma-Poisson distribution has provided a satisfactory fit to RNA sequencing data [24]. A Gamma mixture of Poisson variables gives a negative binomial (NB) distribution [25] which is more appropriate for handling data overdispersion (e.g., microbial count data is highly zero inflated). As a result, we can simulate and generate augmented samples which consists of unnormalized microbial counts. We then use the same preprocessing procedure (described in Acquisition and preprocessing of metagenomic data) to normalize the augmented samples before training our classifiers.

To generate a NB sample, we first assume the mean of the Poisson distribution (*λ*) to be a Gamma-distributed random variable *Γ*(*r*,*θ*) with shape parameter *r* and scale *θ*=*p*/(1−*p*). Note that by construction, the values of *r* and *θ* are greater than zero. Next, we sample the Poisson mean *λ* from this Gamma distribution. Finally, we sample the NB random variable from Pois(*u*;*λ*). The compact form of the mass distribution of a discrete NB random variable (*v*) then reads as:

1 $$\begin{array}{*{20}l} \text{NB}(v; r, p) = \frac{\Gamma(r + v)}{v!\Gamma(r)}p^{v} (1-p)^{r}  \end{array} $$

where *Γ* is the gamma function and the data overdispersion is controlled by the parameter *r*. The NB model reduces to the standard Poisson model for *r*→*∞*. Note that, samples of a given class are assumed to be independent and identically distributed (from one NB distribution). Therefore, we fit a NB distribution for each class. More specifically, we can estimate the model parameters *r* and *θ* using the method of moments. Let *μ*_*i*_ be the mean of OTU *i* and *σ*_*i*_ be the variance of OTU *i*. Note that, the mean and variance of the Gamma distribution is *rθ* and *rθ*^2^, respectively. We can compute the sample mean ($\hat {\mu }$) and sample variance ($\hat {\sigma }$) from the OTU table and then relate them with the model parameter *r* and *θ*. We then arrive at two equations: $\hat {\mu } = r\theta $ and $\hat {\sigma } = r\theta ^{2}$. By solving this two equations, we are able to estimate *r* and *θ* based on the sample mean and sample variance.

### Synthetic data generation

In order to quantitatively evaluate different ML models for classifying microbial samples, we first generate synthetic microbial data that consider multiple sources of measurement errors. More specifically, we first determine the number of classes of interest and then randomly generate the microbiome profile for each class. Next, we sample the microbial count data for each class independently based on the NB distribution and the previously generated microbiome profile. To account for the variability in the real data, we consider three types of errors in measuring the 16S rRNA sequencing data:

- Type 1 error (*e*_1_): the underlying true count is zero (*d*=0) but the measurement count is non-zero ($\hat {d} \neq 0$).
- Type 2 error (*e*_2_): the underlying true count is non-zero (*d*≠0) but the measurement count is zero ($\hat {d} = 0$).
- Type 3 error (*e*_3_): the underlying true count is non-zero (*d*≠0) but with a deviation/fluctuation from the true count ($\hat {d} = d + \text {noise}$).

We generate synthetic data with random combinations of error probabilities [*e*_1_,*e*_2_,*e*_3_]. For example, if *e*_1_=0.5,*e*_2_=0.3,*e*_3_=0.2, we have a probability of 0.5 to add microbial counts to the zero count entries of the underlying true microbial count data. Similarly, for Type 2 and 3 errors, we set the non-zero count to zero with probability of 0.3 and add deviation or fluctuation counts to the non-zero count data with probability of 0.2, respectively.

**Fig. 2:**
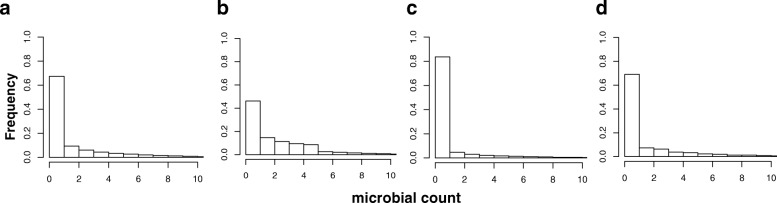
Synthetic microbial frequency count distribution generated using NB distribution based on microbiome profiles. **a** The underlying true distribution which is highly zero inflated (i.e., no presence of certain microbe). **b** Type 1 error that adds non-zero noise to the zero count entries in order to change the distribution. **c** Type 2 error that changes the underlying non-zero entries to zeros. **d** Type 3 error changes the distribution of non-zeros counts. Note that all different types of errors are added with probability of 0.5

As shown in Fig. 2, we can see that three different error types can dramatically change the underlying true count distribution. We evaluate the effects of different combinations of error types on the performance of ML models, as well as multilayer perceptron (MLP) and convolutional neural network (CNN); results are presented later in Experiments with synthetic data.

### MetaNN framework

As shown in Fig. 1, our proposed framework, MetaNN, consists of two important components: First, a new model based on neural networks that is well-suited for classifying metagenomic data. Second, our proposed data augmentation for the microbial count data and adopted dropout training technique that can effectively mitigate the problem of data over-fitting.

#### Multilayer perceptron (MLP)

We consider MLP [26] models with design restrictions on the number of hidden layer and hidden unit in order to prevent over-fitting of the microbial data. To this end, we consider two or three hidden layers where each hidden unit is a neuron that uses a nonlinear activation function; this distinguish MLP from a linear perceptron. Therefore, it is possible to distinguish data that is not linearly separable.

More specifically, MLP uses a supervised learning algorithm that learns a function *f*(·):*R*^*m*^→*R*^*o*^ by training on a dataset, where *m* is the number of input dimensions and *o* is the number of output dimension. Given a set of features *X*=(*x*_1_,*x*_2_,…,*x*_*m*_) and a target *Z*=(*z*_1_,*z*_2_,…,*z*_*o*_), MLP can learn a non-linear function approximator for either classification or regression; this is different from logistic regression, in that between the input and the output layers, there can exist one or more non-linear layers (hidden layers).

**Fig. 3:**
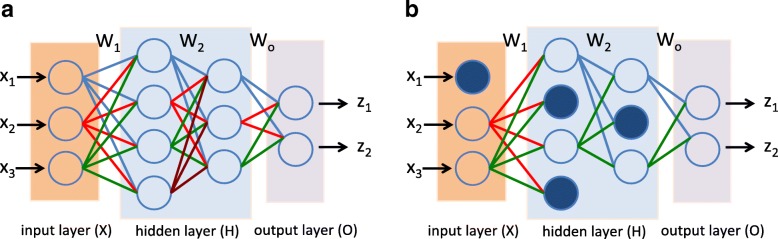
Illustration of random dropout where dropout units are shown as blue filled circles. **a** No dropout. **b** With dropout. As it can be seen, connections to the dropout units are also disabled. Since we randomly choose dropout units in NNs, this means we effectively combine exponentially many different NN architectures to prevent data over-fitting

As shown in Fig. 3a, the leftmost layer, known as the input layer, consists of a set of neurons *X*=(*x*_1_,*x*_2_,*x*_3_) representing the input features. Each neuron in the hidden layer transforms the values from the previous layer with a weighted linear summation *H*_1_=*W*_1_*X*, followed by a non-linear activation function *g*(·):*R*→*R* - like the Rectifier function (i.e., *g*(*x*)=*max*(0,*x*)). The output layer receives the values from the last hidden layer (*H*_2_) and multiplies them with the output weights (*W*_*o*_) hence the output values as *Z*=(*z*_1_,*z*_2_)=*W*_*o*_*H*_2_.

To train the MLP if there exist more than two classes, the output layer is the softmax function which is written as:

2 $$\begin{array}{*{20}l} \hat{z}_{k} = \text{softmax}(z_{k}) = \frac{\exp(z_{i})}{{\sum\nolimits}_{l=1}^{k}\exp(z_{l})}  \end{array} $$

where $\hat {z}_{k}$ represents the estimated probability of having class *k*. Consequently, the predicted label $\hat {y}$ = $\max _{k} \hat {z}_{k}$ is the class with the highest probability. The training objective (loss function) is a cross entropy loss [27] which is represented by:

3 $$\begin{array}{*{20}l} J = -\sum\limits_{i}^{N} \sum\limits_{k}^{K} y^{(i)} \log{\hat{z}^{(i)}_{k}}  \end{array} $$

where *N* is the number of training samples and *K* is the total number of classes. *y*^(*i*)^ is the true class label for sample *i*. $z_{k}^{(i)}$ is the probability of having class *k* for sample *i*.

#### Convolutional neural network (CNN)

The rationale of using CNN to extract local patterns of microbes is that prior studies have found that phylogenetically related microbes interact with each other and form functional groups [28]. Therefore, we arrange the bacterial species based on their taxonomic annotation, ordered alphabetically, by concatenating the strings of their taxonomy (i.e., phylum, class, order, family, and genus). As a consequence, CNN is able to extract the evolutionary relationship based on the phylogenetic-sorting.

**Fig. 4:**
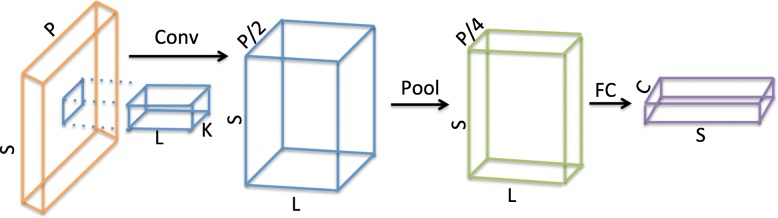
A regular convolutional neural network (CNN). The input consists of *S* samples and *P* features. The 1D filter with kernel size of *K* and *L* channels is used for convolving data with the input. By pooling (downsampling) with kernel size of 2, the resulting tensor now becomes approximately of size *S*×*P*/4×*L*. The fully connected layer considers all the features in every channels and output the probability of class labels (*C*) for each sample

The hidden layers of a CNN typically consist of a set of convolutional layers (Conv), pooling layers (Pool), and fully connected layers (FC) [27]. As shown in Fig. 4, convolutional layer computes the output of neurons that are connected to local regions in the input, each computing a dot product between their weights and a small region they are connected to in the input volume (phylogenetic-sorted). The pooling layer performs a downsampling operation along the spatial dimensions. The fully connected layer computes the class scores which is the same as the output layer of MLP. In our implementation, we consider 1D convolutional and 1D pooling layers since each microbial sample is one dimensional. The training objective is the same as (3).

#### Data augmentation

Data augmentation has been widely used in computer vision communities [8]. For example, in image classification, images are cropped or rotated in order to augment the training set. Data augmentation is useful because it directly augments the input data to the model in data space; this idea can be traced back to augmentation performed on the MNIST set in [29].

Existing metagenomic datasets have fewer samples than the number of observed taxa (features); this makes it difficult to model complex interactions between taxa and differentiate the microbiome profiles [30, 31]. In order to deal with such problems, we propose to augment the microbial data with new samples generated from a known distribution. More specifically, we first use the NB distribution defined in Modeling the microbiome profile to fit the model parameters of the microbiome profile of each class. Next, we use the fitted NB distribution to generate augmented samples for each class. The samples generated by the NB distribution can be viewed as variations in the data space that effectively mitigate the problem of data over-fitting. Note that we only fit the NB distribution to the training set of each split, and then feed both augmented and training datasets to our newly proposed NN classifiers.

#### Dropout

Dropout is a technique proposed to addresses data over-fitting [9], and provides a way of approximately combining exponentially many different neural network architectures efficiently. The term “dropout" refers to temporary dropping out units (hidden and visible) in the NNs, along with all its incoming and outgoing connections, as shown in Fig. 3b.

The choice of which units to drop is random. In the simplest case, each unit is retained with a fixed probability *q* independent of all other units, where *q* can be simply set at 0.5. In our experimental settings, we use dropout at the input layer for both MLP and CNN with a dropout probability of 0.5, which is commonly used and close to optimal for a wide range of networks and tasks [9].

## Results

### Experiments with synthetic data

To show the applicability of MLP and CNN models, we compare our model against several supervised classification ML models (as described in Review of ML methods). This set of experiments serves as a proof of concept of quantifying the performance of each model by simulating synthetic data that account for different levels of measurement error in the real data.

#### Experimental setup

Hyperparameter configurations for MLP and CNN are described in Table 2. To train the model, we use softmax function (Eq. (2)) as the output layer and the cross entropy loss (Eq. (3)) for both MLP and CNN. We implement our MLP and CNN models in Pytorch (http://pytorch.org/) and use Adam [32] as our gradient optimizer with a default learning rate of 0.001 in the subsequent experiments. We fix the training epoch (i.e., one forward and one backward pass over all training instances) to 100 and 200 for MLP and CNN to avoid data over-fitting, respectively. Note that for the synthetic experiments, we do *not* apply any training techniques (i.e., data augmentation and dropout) during model training. The number of hidden units is selected based on the number of feature of the input data. For example, if the number of features is 512 then we choose the number of hidden units in the range of [1024, 512, 256]. The hyperparameters for MLP and CNN are reported in Table 2.

**Table 2:** Model configurations for MLP and CNN

	Synthetic	CBH	CSS	HMP	CS	FS	FSH	IBD	PDX
MLP	(256, 256)	(1024, 512)	(512, 256)	(512, 256)	(512, 512)	(512, 512)	(512, 256)	(512, 256, 128)	(512, 256, 128)
CNN	Conv1D(8, 3) → Dropout → ReLu → MaxPool1D(2) → Conv1D(8, 3) → ReLu → MaxPool1D(2) → FC								

Number in the round bracket represents the number of hidden units. Conv1D is the one-dimensional convolution layer. ReLu is the non-linear rectifier layer. MaxPool1D represents the one-dimensional max pooling layer. Dropout and FC represent dropout and fully connected layers, respectively. Details of each dataset are described in Table 1

For SVM (see Support vector machines (SVMs)), we first select either a linear and radial basis function (RBF, also known as Gaussian kernel) and then select the best regularization parameter and width parameter in the range of [10^−2^,…,10^2^,10^3^] and [10^−5^,…,10^1^], respectively, using a 3-fold cross-validation approach. For GB (see Gradient boosting (GB)), we set up a higher maximum depth equal to 10; minimum samples split equal to 5 as a compromise between over-fitting and under-fitting the training set. For RF (see Random forests (RF)), we set up the number of estimators equal to 200 (default is 10) to have a better estimation and then select the depth, sample splits, and number of leaves using 3-fold cross-validation. For MNB (see Multinomial naïve bayes (MNB)), we fit a prior distribution to the number of OTUs in each class; this acts as a smoothing constant. For other ML methods and hyperparameters, we use the default values implemented in *scikit-learn*.

#### Classification performance metrics

We consider a few metrics as follows:

- Area under the Curve (AUC): We compute the area under receiver operating characteristic (ROC) curve where a larger area means a better classification model.
- F1-micro: We estimate F1-micro as the true positives plus the true negatives divided by the total number of samples; this is same definition of classification accuracy as widely used in binary classification problems.
- F1-macro: We estimate F1-macro by calculating the F1-micro for each class and then find their unweighted mean; this does not take label imbalance into account.
- Performance Gain: We calculate the performance gain as the F1 score of the best NN model minus the F1 score of the best ML models divided by the F1 score of the best ML models.

#### Classification performance comparisons

We consider eight classes each with different microbiome profiles (the generation process of synthetic data is discussed in Synthetic data generation). For example, consider the case when the number of microbes is *p*=100 for each class. For a particular microbiome profile (e.g., *m*=(30,40,30) microbes), we sample three different overdispersion parameters (e.g., *r*=(0.1,1,10)) for the NB distribution, respectively. Next, we use *r* and sample the microbial counts based on Eq. (1) and then alter the counts by adding different sources of errors with specific probabilities.

We report the results for eight classes where each class has *d*=100 samples and *p*=100 microbes. As shown in Table 3, when we fix the probability of Type 1 errors (*e*_1_) to 0.5 and 0.0 and vary the probability of Type 2 (*e*_2_) and Types 3 (*e*_3_) errors, we find that the Type 3 errors are more severe than the Type 2 errors; this is because the Type 3 errors can dramatically change the microbial count distribution as shown in Fig. 2. We also find that the Type 1 errors have a moderate impact on the performance of each classifier.

**Table 3:** Performance comparison of different ML and NN models for different types of error (*e*_1_,*e*_2_,*e*_3_)

(*e*_1_,*e*_2_,*e*_3_)	SVM	GB	RF	MNB	LR1	LR2	MLP	CNN
F1-micro								
(0.5, 0.1, 0.4)	0.96	0.79	**0.98**	**0.98**	0.30	**0.98**	0.98	0.75
(0.5, 0.4, 0.1)	0.99	0.82	**1.00**	**1.00**	0.43	**1.00**	**1.00**	0.81
(0.3, 0.1, 0.4)	0.98	0.87	0.98	**0.99**	0.54	**0.99**	**0.99**	0.74
(0.0, 0.7, 0.2)	0.99	0.83	**1.00**	**1.00**	0.66	**1.00**	**1.00**	0.86
(0.0, 0.2, 0.7)	0.89	0.58	0.81	**0.91**	0.51	0.87	**0.91**	0.59

We consider several existing supervised ML methods, as well as NN models (i.e., MLP and CNN). For each experiment, we use 10-fold cross-validation. We use F1-micro to quantify the performance as defined in Classification performance metrics. Bold values represent the best results

We find that MLP and MNB achieve the best (and comparable) performance in all scenarios we considered; this is due to the fact that MLP is able to better deal with the sparse features since NNs can extract higher level features by utilizing hidden units in hidden layers. MNB fits the prior distribution for the microbiome profile of each class; this can largely improve performance since each class is generated based on the NB distribution which complies with the underlying assumptions of MNB. Overall, MLP is suitable to deal with different sources of errors. On the contrary, CNN is not able to deal with sparse features since the convolution layer considers spatial relationships among features; this results in its poor performance for the synthetic datasets.

### Experiments on real data

We utilize several datasets (see Acquisition and preprocessing of metagenomic data) to examine the performance of different ML models in real scenarios. Datasets can be classified into three categories based on their properties: (1) Classification of body sites, (2) classification of subjects, and (3) classification of disease states. The total number of samples and features (i.e., OTUs) are summarized in Table 1. We also list the model hyperparameters for MLP and CNN in Table 2. In our experimental settings, the number of augmented samples is set equal to the number of training samples, the dropout rate (*q*) is set to 0.5. We use the same set of hyperparameters for the other ML methods, as described in Section 1.

#### Performance of ML models on real data

The performance of all the ML methods introduced in Review of ML methods is summarized in Table 4. As it can be seen, SVM and RF have better performance compared to other remaining methods in terms of F1-score. Since SVM and RF have better performance over other ML methods, we choose these two methods to compare with our NN models in Table 5.

**Table 4:** Performance comparison of ML models on eight real datasets described in Table 1

Dataset	SVM	RF	GB	MNB	LR1	LR2
F1-macro						
CBH	0.78(0.03)	0.73(0.03)	0.74(0.04)	0.66(0.03)	0.41(0.04)	0.17(0.01)
CSS	0.63(0.07)	0.58(0.08)	0.48(0.05)	0.49(0.03)	0.26(0.03)	0.24(0.02)
HMP	0.97(0.01)	0.97(0.01)	0.95(0.01)	0.95(0.01)	0.94(0.01)	0.93(0.01)
CS	0.88(0.05)	0.87(0.05)	0.74(0.06)	0.76(0.04)	0.16(0.04)	0.19(0.06)
FS	0.94(0.03)	1.00(0.01)	0.91(0.06)	0.98(0.01)	0.60(0.05)	0.58(0.04)
FSH	0.68(0.04)	0.63(0.08)	0.55(0.06)	0.50(0.04)	0.17(0.01)	0.17(0.00)
IBD	0.68(0.04)	0.57(0.02)	0.65(0.02)	0.43(0.01)	0.47(0.02)	0.43(0.01)
PDX	0.29(0.13)	0.28(0.09)	0.35(0.05)	0.18(0.03)	0.15(0.01)	0.15(0.01)
F1-micro						
CBH	0.93(0.02)	0.91(0.02)	0.89(0.02)	0.88(0.02)	0.76(0.02)	0.68(0.00)
CSS	0.71(0.03)	0.67(0.03)	0.57(0.04)	0.58(0.03)	0.48(0.03)	0.48(0.03)
HMP	0.97(0.01)	0.97(0.01)	0.95(0.01)	0.95(0.01)	0.94(0.01)	0.93(0.01)
CS	0.88(0.06)	0.88(0.04)	0.75(0.05)	0.75(0.05)	0.23(0.05)	0.28(0.07)
FS	0.94(0.03)	1.00(0.01)	0.91(0.06)	0.98(0.01)	0.68(0.03)	0.67(0.03)
FSH	0.70(0.08)	0.69(0.05)	0.58(0.06)	0.62(0.03)	0.33(0.01)	0.33(0.01)
IBD	0.79(0.02)	0.78(0.02)	0.77(0.02)	0.76(0.02)	0.76(0.02)	0.76(0.02)
PDX	0.44(0.07)	0.43(0.07)	0.40(0.05)	0.42(0.04)	0.42(0.04)	0.42(0.04)

We consider several existing supervised ML methods. For each experiment, we consider 10-fold cross-validation and use F1-macro and F1-micro scores to quantify performance as defined in Classification performance metrics. For each fold, we perform five simulation runs with standard deviations shown between round brackets

**Table 5:** Performance comparison of SVM, RF and NN models on eight real datasets described in Table 1

Dataset	SVM	SVM+A	RF	RF+A	MLP+D	CNN+D	MLP+D+A	CNN+D+A	Gain (%)
**F1-macro**									
CBH	0.78 (0.03)	0.82 (0.03)	0.73 (0.03)	0.75 (0.03)	0.85 (0.03)	0.77 (0.04)	0.86 (0.03)	0.82 (0.03)	5
CSS	0.63 (0.07)	0.65 (0.06)	0.58 (0.08)	0.61 (0.06)	0.66 (0.06)	0.59 (0.06)	0.67 (0.06)	0.62 (0.06)	3
HMP	0.97 (0.01)	0.97 (0.01)	0.97 (0.01)	0.97 (0.01)	0.97 (0.01)	0.97 (0.01)	0.97 (0.01)	0.97 (0.01)	0
CS	0.88 (0.05)	0.88 (0.05)	0.87 (0.05)	0.87 (0.05)	0.92 (0.05)	0.87 (0.06)	0.93 (0.05)	0.88 (0.05)	6
FS	0.94 (0.03)	0.95 (0.02)	1.00 (0.01)	1.00 (0.01)	0.97 (0.03)	0.90 (0.15)	0.98 (0.02)	0.97 (0.02)	-2
FSH	0.68 (0.08)	0.70 (0.08)	0.63 (0.08)	0.68 (0.08)	0.74 (0.06)	0.66 (0.07)	0.74 (0.05)	0.72 (0.07)	6
IBD	0.68 (0.04)	0.72 (0.02)	0.57 (0.02)	0.60 (0.02)	0.75 (0.02)	0.67 (0.03)	0.78 (0.02)	0.70 (0.02)	8
PDX	0.29 (0.13)	0.43 (0.02)	0.28 (0.09)	0.34 (0.07)	0.51 (0.00)	0.44 (0.05)	0.56 (0.03)	0.45 (0.08)	30
**F1-micro**									
CBH	0.93 (0.02)	0.93 (0.01)	0.91 (0.02)	0.92 (0.02)	0.94 (0.01)	0.89 (0.02)	0.94 (0.01)	0.92 (0.02)	1
CSS	0.71 (0.03)	0.72 (0.04)	0.67 (0.03)	0.68 (0.03)	0.72 (0.03)	0.67 (0.04)	0.74 (0.03)	0.68 (0.04)	3
HMP	0.97 (0.01)	0.97 (0.01)	0.97 (0.01)	0.97 (0.01)	0.97 (0.01)	0.96 (0.01)	0.97 (0.01)	0.97 (0.01)	0
CS	0.88 (0.06)	0.89 (0.05)	0.88 (0.04)	0.88 (0.05)	0.92 (0.04)	0.87 (0.06)	0.94 (0.04)	0.89 (0.05)	6
FS	0.94 (0.03)	0.95 (0.02)	1.00 (0.01)	1.00 (0.01)	0.97 (0.03)	0.91 (0.12)	0.98 (0.02)	0.97 (0.02)	-2
FSH	0.70 (0.08)	0.71 (0.07)	0.69 (0.05)	0.72 (0.06)	0.75 (0.05)	0.68 (0.06)	0.76 (0.05)	0.75 (0.07)	6
IBD	0.79 (0.02)	0.79 (0.02)	0.78 (0.02)	0.79 (0.02)	0.82 (0.01)	0.77 (0.02)	0.84 (0.01)	0.78 (0.02)	6
PDX	0.44 (0.07)	0.48 (0.03)	0.43 (0.07)	0.44 (0.06)	0.53 (0.01)	0.49 (0.05)	0.56 (0.03)	0.50 (0.06)	17

+D and +A means dropout and data augmentation, respectively. For each experiment, we consider 10-fold cross-validation and use F1-macro and F1-micro scores to quantify performance as defined in Classification performance metrics. For each fold, we perform five simulation runs with standard deviations shown between round brackets. Performance gains are shown for the best NN and the best ML models. Bold values show the best results

**Fig. 5:**
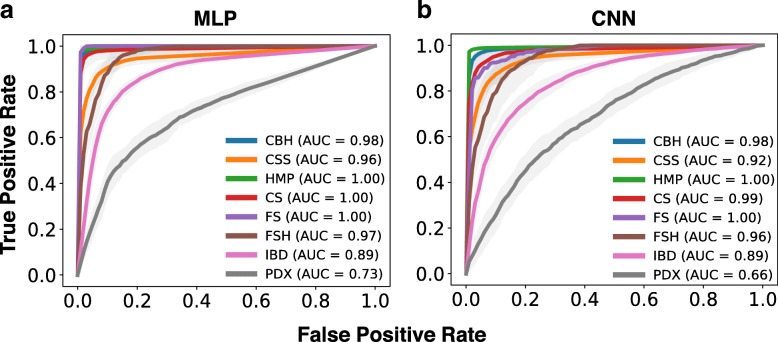
ROC curves and AUCs for (**a**) multilayer perceptron (MLP) and (**b**) convolutional neural network (CNN). True positive rates are averaged over 10-fold cross-validation each with 5 independent random runs. We show the ROC curves and AUCs for the real datasets considered in this paper

We first show the classification performance of MLP and CNN on different datasets using ROC curves. As shown in Fig. 5, MLP shows better performance than CNN; this implies that MLP is a better model since the activation function at the output layer is able to learn a better decision boundary. Additionally, we find that disease datasets (i.e., IBD and PDX) are more difficult to classify. In the following sections, we present the experiment results for datasets in different categories.

#### Classification of body sites

In this set of experiments, we consider a total of three datasets: two came from [20] and one from HMP (see Table 1). As discussed in [5] and shown in Table 5 and Fig. 5, CSS is the most difficult dataset since the microbiome profiles are generally non-differentiable between different skin sites. For the other two datasets (i.e., CBH and HMP), the microbiome profiles tend to be highly differentiated between different body sites; therefore, ML models do obtain a better classification performance. In practice, classification of body sites would not require the use of a predictive model for classification since we would most likely know the site of sampling. However, it is still valuable to use this category to evaluate the performance of different ML methods.

#### Classification of subjects

In this set of experiments, we consider three benchmark datasets where two come from [21] and one from [20]. As shown in Table 5 and Fig. 5, this category is more challenging than classifying body sites since the samples of certain subject may be collected at different time points. For the CS dataset, authors in [20] observed significant variations of microbiome profile for individuals over time and most ML models cannot achieve a high accuracy. On the contrary, for the FS dataset, individuals have clear differences since samples are collected at approximately the same time point. FSH dataset is more challenging compared to FS since we need to additionally classify the right and left hand for each individual.

#### Classification of disease states

In this set of experiments, we consider IBD and PDX datasets from [11] and [12], respectively. As shown in Tables 1 and 5, PDX is a challenging dataset, since it contains four classes and the microbiome profiles are similar among these classes. Indeed, existing ML models can only achieve up to 40% accuracy (F1-micro score) of the PDX set.

#### Classification performance comparisons

As shown in Table 5, MLP with dropout and data augmentation (MLP+D+A) achieves the best performance in terms of F1-macro and F1-micro scores among all other ML methods, except the FS dataset. CNN with dropout and data augmentation (CNN+D+A) also provides comparable performance with other ML models. Note that without using data augmentation, MLP (MLP+D) still achieves the best performance against other ML models; this is because MLP can extract higher level features and automatically select the important features.

Other than MLP and CNN, SVM and RF also show better performance; this is because SVM and RF are able to distinguish features even in high dimensional settings while being robust to random features. However, MLP can still have significant average gains of 7% and 5% against the best ML method in terms of F1-macro and F1-micro, respectively. If we take a closer look at the disease datasets, we can see that the MLP+D+A has a dramatic increase in terms of F1-macro scores (8% and 30% gains) compared to other ML methods for both IBD and PDX datasets; this indicates that MetaNN can accurately differentiate and better classify various disease states.

As shown in Table 5, data augmentation can improve the classification performance not only for NN models but also for ML models. More specifically, we can have an average of 2-3% improvement compared to the one without using data augmentation; this shows that data augmentation in the training sets can truly leverage the high dimensionality of metagenomic data.

In terms of classification performance of ML methods listed in Table 5, we can see that ML methods can achieve up to 80-100% F1 scores for most of the datasets. For example, both MLP and RF can achieve up to 98% classification accuracy for the FS dataset. However, other challenging datasets, such as PDX and CSS have non-differentiable microbiome profiles. To support this claim, we utilize the (1) Q-Q (quantile-quantile) plot to quantify two distributions against each other, and (2) scatter plot to show the consistency of microbiome profiles between different classes.

**Fig. 6:**
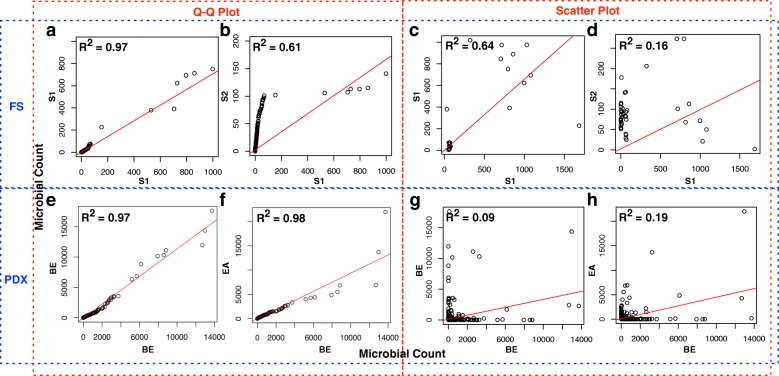
(**a**-**b** and **e**-**f**) Q-Q plots and (**c**-**d** and **g**-**h**) scatter plots for FS and PDX datasets, respectively. The red line is the linear fitted line with adjusted R square reported at the top-left corner. S1, S2 represent samples from subject 1 and subject 2, respectively. BE, EA represent samples from Barrett’s esophagus (BE) and esophageal adenocarcinoma (EA) patients, respectively

Q-Q plot is generated based on the quantiles of two distributions, where quantile can be obtained by sorting the microbial counts. For example, Fig. 6b shows the quantile distributions of subject 1 (S1) against subject 2 (S2). On the contrary, the scatter plot is generated based on the (unsorted) microbiome profile. For example, a point on Fig. 6d represents a certain microbe (e.g., E. coli) found in both S1 and S2 samples but with different counts.

For the FS dataset, we first notice that subject 1 (S1) within-class distribution and profile are similar (Fig. 6a, c) as opposed to between-class case (Fig. 6b, d); these distinct differences make the FS dataset easy to classify. However, for the PDX dataset, we can see that the distribution and profiles of PDX dataset show completely different behaviors compared to the FS dataset. Microbiome distributions and profiles for Barrett’s esophagus (BE) and esophageal adenocarcinoma (EA) patients are shown to be very similar (adjusted R squares up to 0.97). Additionally, the scatter plots (profiles) also show that BE and EA profiles (Fig. 6g, h) are more similar than samples from BE (Fig. 6e, g). As a consequence, ML models are unable to distinguish these two classes which results in their poor performance.

#### Neural network visualization

**Fig. 7:**
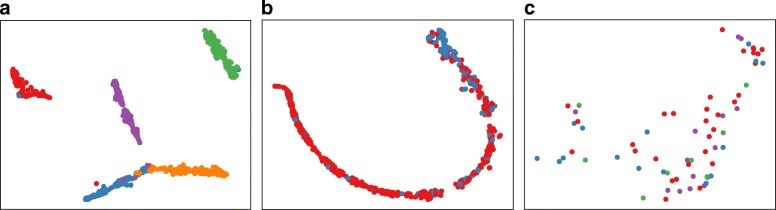
Visualization of (**a**) HMP, (**b**) IBD, and (**c**) PDX datasets using t-SNE projection [33]. We project the activation function of the last hidden layer of the test data onto a 2D space, where different colors represent different classes. For instance, the red and green colors represent samples collected from anterior nares and stools, respectively. As it can be seen, HMP and IBD samples show a clear separation between classes, while PDX samples are hard to be distinguished

Visualization of the last hidden layer of the test data can further show that neural network can learn meaningful feature representations. By projecting the activation function of the last hidden layer using t-SNE [33] on a two-dimensional space, we can observe there are obvious distinctions among different classes for HMP and IBD datasets (see Fig. 7a, b); this shows that neural network provides a non-linear transformation of data that can identify different body sites and subjects diagnosed with IBD. However, for the PDX dataset, there is no clear distinction between different classes which results in poor performance for every ML-based classifiers.

## Discussion

Advances of high-throughput sequencing techniques enable researchers to gather metagenomic data from different environment and human niches. The available high-throughput experimental data, however, are high-dimensional in nature; this makes it challenging for researchers to identify and disentangle the underlying microbiome profiles that relate to different human phenotypes such as body sites and disease states.

Although several existing ML models have been proposed for classifying metagenomic data, their performance is mostly unsatisfactory. To boost the classification accuracy, we have proposed a new neural network based pipeline that is suitable for classifying metagenomic datasets. However, the high-dimensional nature and limited number of microbial samples can make such models easily over-fit the training set and thus result in poor classification of new samples. To remedy the data over-fitting problem, we have proposed data augmentation and dropout during training.

Our analysis on real datasets has revealed that ML methods can achieve high classification accuracy when datasets have distinct distributions among different classes. On the contrary, challenging datasets like PDX show similar distributions for different classes; therefore, the existing ML classifiers are unable to distinguish in such situations, while our proposed MetaNN has significant improvements on the classification accuracy. Ultimately, an ideal classifier needs good feature selection mechanisms to select a subset of features that is the most representative for a particular class. In this respect, NNs are well-suited for automatic feature selection and engineering; this makes NNs better than other ML models for classifying metagenomic data.

Experimental results show that the new data augmentation can effectively improve the classification performance for both NN models and ML models. More importantly, when using the augmented training set, the classification results are as good as or better than that of the best non-augmented model; this shows that data augmentation can truly leverage the high dimensionality of metagenomic data and effectively improve the classification accuracy.

## Conclusion

In this paper, we have shown that our proposed MetaNN outperforms all other existing methods for both synthetic and real data. For the synthetic experiments, we have evaluated several combinations of measurement errors to demonstrate the applicability of MetaNN to different conditions. For real datasets, our MetaNN has average gains of 7% and 5% in terms of F1-macro and F1-micro scores, respectively. Overall, MetaNN has shown very promising results and better performance compared to existing ML methods.

## Abbreviations

AUC: Area under the receiver operating characteristics curve
CNN: Convolutional neural network
GB: Gradient boosting
HMP: Human microbiome project
IBD: inflammatory bowel disease
LR: Logistic regression
ML: Machine learning
MLP: Multilayer perceptron
MNB: Multinomial naïve bayes
NB: Negative binomial
NN: Neural network
OTU: Operational taxonomic units
PCoA: Principal coordinates analysis
RF: Random forest
ROC: Receiver operating characteristics
SVM: Support vector machines
T2D: Type 2 diabetes

## References

[1] Halfvarson J, et al.Dynamics of the human gut microbiome in inflammatory bowel disease. Nat Microbiol. 2017;2. https://www.nature.com/articles/nmicrobiol20174. 10.1038/nmicrobiol.2017.4 PMC5319707 28191884

[2] Zhang Y, Zhang H (2013). Microbiota associated with type 2 diabetes and its related complications. Food Sci Hum Wellness.

[3] Anderson MJ, Willis TJ (2003). Canonical analysis of principal coordinates: A useful method of constrained ordination for ecology. Ecology.

[4] Statnikov A (2013). A comprehensive evaluation of multicategory classification methods for microbiomic data. Microbiome.

[5] Knights D (2011). Supervised classification of human microbiota. FEMS Microbiol Rev.

[6] Consortium THMP (2012). Structure, function and diversity of the healthy human microbiome. Nature.

[7] Breiman L (2001). Random forests. Mach Learn.

[8] Krizhevsky A (2012). Imagenet classification with deep convolutional neural networks. Proceedings of the 25th International Conference on Neural Information Processing Systems - Volume 1. NIPS’12.

[9] et al., NS. Dropout: A simple way to prevent neural networks from overfitting. J Mach Learn Res. 2014; 15:1929–58.

[10] McMurdie PJ, Holmes S (2014). Waste not, want not: Why rarefying microbiome data is inadmissible. PLoS Comput Biol.

[11] Gevers D (2011). The treatment-naive microbiome in new-onset crohn’s disease. Cell Host Microbe.

[12] Yang L, et al. In: Nelson KE, editor. Foregut Microbiome, Development of Esophageal Adenocarcinoma, Project. New York: Springer: 2013. p. 1–5.

[13] Rijsbergen CJV (1979). Information Retrieval.

[14] Chang Chih-Chung, Lin Chih-Jen (2011). LIBSVM. ACM Transactions on Intelligent Systems and Technology.

[15] Fan R-E (2008). Liblinear: A library for large linear classification. J Mach Learn Res.

[16] Friedman JH (2000). Greedy function approximation: A gradient boosting machine. Ann Stat.

[17] Manning CD (2008). Introduction to Information Retrieval.

[18] Furey TS (2000). Support vector machine classification and validation of cancer tissue samples using microarray expression data. Bioinformatics.

[19] Hastie T, et al.The Elements of Statistical Learning: Data Mining, Inference and Prediction, 2nd edn.: Springer; 2009.

[20] Costello EK (2009). Bacterial community variation in human body habitats across space and time. Science.

[21] Fierer N (2010). Forensic identification using skin bacterial communities. Proc Natl Acad Sci.

[22] Kuczynski J, Stombaugh J (2012). Using QIIME to analyze 16S rRNA gene sequences from Microbial Communities. Curr Protoc Bioinforma.

[23] Lu J (2005). Identifying differential expression in multiple sage libraries: an overdispersed log-linear model approach. BMC Bioinformatics.

[24] Robinson MD, Smyth GK (2008). Small-sample estimation of negative binomial dispersion, with applications to sage data. Biostatistics.

[25] et al., MZ. Beta-negative binomial process and poisson factor analysis. Proc Fifteenth Int Conf Artif Intell Stat. 2012; 22:1462–71.

[26] Hinton GE (1989). Connectionist learning procedures. Artif Intell.

[27] Goodfellow I, et al.Deep Learning: MIT Press; 2016.

[28] Faust K, Sathirapongsasuti J (2012). Microbial co-occurrence relationships in the human microbiome. PLoS Comput Biol.

[29] Baird HS (1992). Structured Document Image Analysis.

[30] Lo C, Marculescu R. Inferring microbial interactions from metagenomic time-series using prior biological knowledge. In: Proceedings of the 8th ACM International Conference on Bioinformatics, Computational Biology,and Health Informatics. ACM-BCB ’17. New York: ACM: 2017. p. 168–77. 10.1145/3107411.3107435. http://doi.acm.org/10.1145/3107411.3107435.

[31] Lo Chieh, Marculescu Radu (2017). MPLasso: Inferring microbial association networks using prior microbial knowledge. PLOS Computational Biology.

[32] Kingma DP, Ba J. Adam: A method for stochastic optimization. CoRR. 2014; abs/1412.6980. http://arxiv.org/abs/1412.6980.

[33] van der Maaten L, Hinton G (2008). Visualizing data using t-SNE. J Mach Learn Res.

